# Results of arthroscopic microfracture treatment for traumatic and non-traumatic osteochondral lesions of the talus: a retrospective cohort study

**DOI:** 10.1186/s12891-025-08949-6

**Published:** 2025-07-25

**Authors:** Muhammed Taha Demir, Yigit Kultur, Hilmi Karadeniz

**Affiliations:** 1 OrthoCure Clinic, Istanbul, Turkey; 2 Department of Ortopaedics and Traumatology, Yeni Yuzyil University Gaziosmanpasa Hospital, Istanbul, Turkey; 3 Department of Physiotherapy, Aydin University Health Sciences, Aydin, Turkey

**Keywords:** Microfracture, Osteochondral lesion, Talus

## Abstract

**Background:**

This study is designed to assess the extent to which the outcomes of arthroscopic microfracture surgery for talus osteochondral lesions (OLTs)—whether of traumatic or atraumatic origin—are influenced by these underlying etiologic factors. Toward this end, it aims to optimise patient selection and treatment plans, thereby enabling the prediction of surgical prognosis.

**Methods:**

This retrospective study included 70 ankles from 70 patients with OLTs, who were treated with microfracture procedures using anterior ankle arthroscopy by orthopaedic surgeons at two different medical centres. Of these cases, 38 were of a traumatic origin (Group 1) and 32 were of a non-traumatic origin (Group 2). The inclusion criteria were adult patients with unilateral, detached and/or displaced lesions located in the medial central region of the talus. Preoperative and final follow-up American Orthopaedic Foot and Ankle Society (AOFAS) and Visual Analogue Scale (VAS) pain scores were compared within and between groups. The rate of return to baseline activity levels was also compared between the various groups. The potential influence of body mass index (BMI) on both etiology and surgical outcome was examined.

**Results:**

The median follow-up period, as the interquartile range, for all patients was 39 months, ranging from 26 to 54.25 months. Both groups of patients showed significant improvement in their AOFAS and VAS scores postoperatively compared with their preoperative assessment (*p* < 0.001). Nevertheless, no statistically significant difference was found in the median AOFAS and VAS scores between Group 1 and Group 2 (*p* > 0.05). After the operation, 66 patients, representing 94.3%, successfully resumed their previous lifestyle, with no difference observed between the two groups (*p* = 0.392). In addition, the mean BMI in Group 2 was significantly higher than in Group 1 (*p* = 0.0035).

**Conclusion:**

Arthroscopic microfracture treatment provides similar clinical outcomes in the case of non-traumatic and traumatic OLTs. A high BMI, however, has been recognized as a significant risk factor for the development of non-traumatic OLTs.

## Introductıon

Unless they are managed effectively, talar osteochondral lesions (OLTs) can lead to significant productivity loss and, due to their progressive nature, contribute to the development of ankle osteoarthritis [1–3]. These lesions can appear acutely following foot and ankle injuries or can develop insidiously as a consequence of non-traumatic forces [3, 4]. Traumatic etiology accounts for about 80% of the lesions, and trauma history was reported in 93% of patients with lateral OLTs and in 62% of patients with medially located lesions [2, 5, 6]. It has been estimated that the development of OLTs occurs in approximately 50% of the events of ankle distortion trauma and in more than 70% of the events of fracture in the ankle [7]. Repeated exposure to microtraumas in patients with signs of foot and ankle instability has been associated with the development of osteochondral lesions in approximately 39% of cases [8]. OLTs predominantly affect young and active males (70%) [9]. With decreased mobility in older adults, the rate of trauma and related OLTs also decreases [10]. Non-traumatic OLTs have been related to avascular necrosis, heredity, endocrine and metabolic disease, microtraumas, and ischemic disease [11].

OLTs usually present clinically with localized pain that is exacerbated by joint use, often with associated swelling and stiffness. Some patients also report a feeling of catching or jamming in the joint during motion. With chronic presentations, the severity of deep pain becomes more pronounced [3, 4, 12]. The most widely used imaging method is plain radiography, which serves to evaluate bone pathology and joint alignment; however, its sensitivity in detecting OLTs is limited, as it misses up to 40% of lesions [9, 13]. Magnetic resonance imaging (MRI) is the preferred diagnostic tool for the diagnosis of osteochondral lesion due to its ability to better evaluate the cartilage, soft tissues, and bone marrow edema [13, 14]. Computed tomography (CT) is superior to MRI in lesion localization and preoperative lesion size evaluation [14, 15].

Asymptomatic OLTs do not require treatment; however, periodic monitoring is recommended because of their potential for progression [1]. In cases of stable and non-displaced symptomatic lesions, conservative management is indicated [9]. Conservative treatments include unloading, activity modifications, non-steroidal anti-inflammatory drugs (NSAIDs), the use of orthoses or a brace, intra-articular injections of steroids or platelet-rich plasma (PRP), and physical therapy modalities [5]. Conservative methods provide successful results in approximately 45–50% of cases [1, 16]. Surgery is indicated for chronic symptomatic OLTs that are refractory to conservative measures, are unstable, or become displaced. The expectation of early return to sports in athletes, as well as the frequency of ankle instability, raises the level of urgency for surgical repair [3, 4, 16–18]. In general, as the lesion size increases, the need for surgical treatment increases [16, 19]. Surgical options include the fixation of large osteochondral fragments, autologous osteochondral transplantation, allografting, autologous chondrocyte implantation, acellular scaffold implantation, and metal resurfacing arthroplasty.

The microfracture method is a technique of bone marrow stimulation performed during arthroscopy, involving the debridement of worn hyaline cartilage and the underlying subchondral bone, followed by the creation of small perforations in the bone. This allows the migration of mesenchymal stem cells from the subchondral bone to the defect area, where the cells differentiate into chondrocyte-like cells and form new tissue composed of type II collagen [20]. This method has demonstrated considerable effectiveness over both short-term and long-term follow-up periods and is widely used in practice [21–23]. The technique is considered the gold standard for treating lesions smaller than 150 mm² [3, 11, 12, 18, 23–25]. As the lesion size gets larger, however, the effectiveness of microfracture treatment decreases significantly [10, 11, 24, 26]. The goal of this procedure is to create a load-bearing surface composed of cartilage-like tissue that is rich in proteoglycans and type II collagen [20]. However, the newly formed tissue is fibrocartilaginous and has inferior biomechanical properties compared to the original hyaline cartilage [3]. Arthroscopic microfracture presents several advantages over open surgical methods, including a lower incidence of complications, greater cost efficiency, and less postoperative pain.

### Classification

In our study, we used the MRI-based classification developed by Hepple and colleagues, which is widely used for the evaluation of OLTs [27]. To determine the topographical location of the lesion, the talar grid system was used, subdividing the talus into nine zones [28]. This classification has the centromedial zone (zone 4) as the most frequently involved area, accounting for about 53% of all OLTs [28]. The centrolateral zone (zone 6) has been recognized as the second most involved area [28]. For the present work, the Hepple MRI classification was used to grade the lesions [27]. For the sake of homogeneity, only patients with lesions in the centromedial zone (zone 4), as defined by the 9-zone system, were included in the present investigation.

This study aims to evaluate the outcomes of bone marrow stimulation through arthroscopic microfracture in patients with OLTs of both traumatic and non-traumatic origins and to assess the impact of prior trauma on treatment results. It also seeks to contribute to the optimisation of patient selection criteria and the prediction of surgical outcomes.

## Materials and methods

**Fig. 1:**
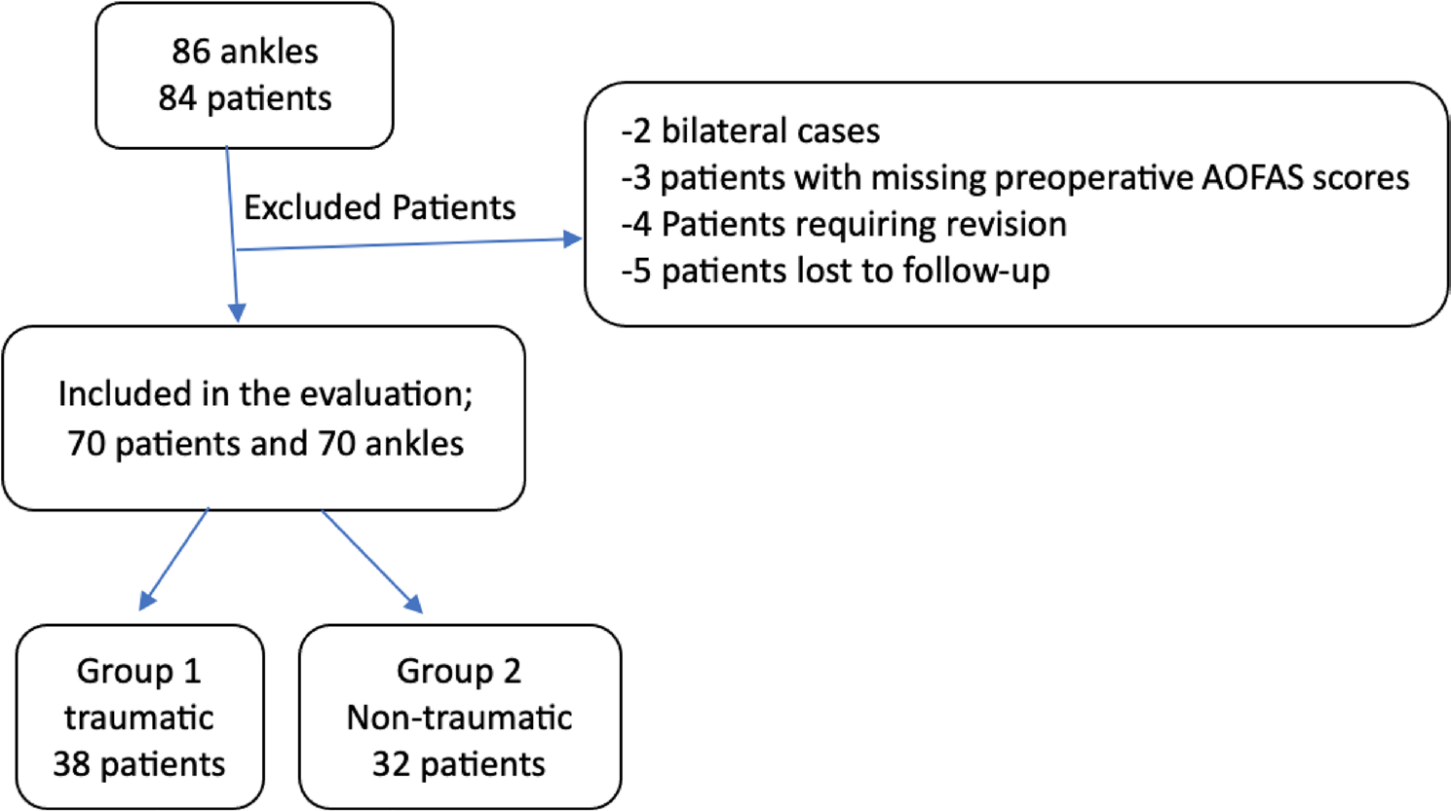
Study participants’ characteristics included in the study. AOFAS: American Orthopaedic Foot and Ankle Society

A retrospective review was performed, including a total of 86 ankles from 84 patients with centromedial zone 4 OLTs, who were treated with bone marrow stimulation through arthroscopic microfractures between the years 2013 and 2024. The current study adhered to the ethical principles outlined in the Declaration of Helsinki, as approved by the Ethics Committee of the institution (Yeni Yuzyil University Clinical Research Ethics Committee, approval number: 2025/04-1530). Informed consent was obtained from patients involved in the study at the time of their final follow-up, prior to participation. Of these cases, 3 patients were excluded because of missing preoperative scores, 4 were excluded because they underwent revision surgery A repeat arthroscopic bone marrow stimulation intervention was performed in two patients after the failure of the first intervention. In another patient, a mosaicplasty was then performed after the initial intervention failed. At the same time, a fourth patient required plate osteosynthesis due to a lateral malleolar fracture after an ankle distortion. Five patients were unable to return for the last follow-up, and two patients presented with bilateral OLTs. Therefore, the analysis was restricted to 70 ankles from 70 patients. Of these, 38 patients underwent surgery for ankle distortion trauma (Group 1), while 32 patients underwent surgery for non-traumatic lesions (Group 2) (Fig. 1).

The preoperative scores of the American Orthopaedic Foot and Ankle Society (AOFAS) and the Visual Analog Scale (VAS) scores were noted in all the subjects of the study. Evaluation of the scores was done at the final follow-up assessment. Patients with zone 4 lesions were included in the study based on preoperative MRI evaluation. Additionally, participants were questioned about the resumption of preoperative activity levels. Before the surgery, body mass index (BMI) was calculated and correlated with the intrinsic etiological factors. At the final follow-up assessment, various symptoms such as pain, stiffness, swelling, catching sensations, and functional loss were assessed, along with observations of deformities and signs of instability.

### Inclusion criteria

- Age > 18 years.
- Lesion size < 1.5 cm².
- A lesion in the centromedial area.
- Received anterior ankle arthroscopy.

### Exclusion criteria

- Previous surgical procedures were performed on the same ankle.
- Presence of instability at the final follow-up.
- Indicators of widespread arthritic changes.
- Identification of a fracture of the ankle on initial examination.

### Surgical procedure

**Fig. 2:**
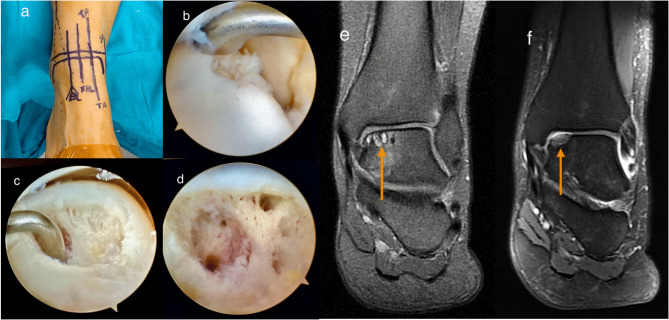
Arthroscopic debridement along with the use of the microfracture technique in non-traumatic OLTs. **a** Preoperative preparation for arthroscopy, **b** Identification of the region of the OLTs using a probe. **c** Post-debridement appearance of the OLTs area, **d** Post-microfracture appearance of the OLTs area, **e** Pre-operative MRI related to the lesion, **f** Postoperative 36-months MRI showing the defect area filled with fibrocartilaginous tissue

All surgeries were performed under either spinal or general anaesthesia. The patient was positioned supine, with the foot about 10 cm from the operating table. A support device was placed under the ipsilateral hip to keep the foot in a neutral position. A tourniquet was applied, but no ankle distractor was used during surgery. Arthroscopy of the ankle joint proceeded through the traditional anterolateral and anteromedial portals. Entrance to the joint allowed visualization of intra-articular structures. Visualisation with a probing instrument facilitated the evaluation of the OLTs according to their grading, which was documented accordingly. Unstable cartilage was debrided with a curette until a well-supported and healthy cartilage remained. All loose tissue was debrided. Subchondral sclerotic and necrotic bone were cleared using a curette and a burr. Measurement of the lesion size was facilitated with the probe tip, and arthroscopic surgery proceeded only for lesions smaller than 1.5 cm². The microfracture technique was performed using an awl with a 60-degree beak and a cone tip to create holes to a depth of approximately 3–4 mm, spaced 3–4 mm apart, thereby keeping the holes separate. Emergence of fat droplets through the holes was checked to ensure the depth attained was adequate (Fig. 2). Patients were instructed to avoid weight-bearing in the postoperative period for the first 6 weeks. Early mobilization was commenced on the same day with active and passive exercises for the range of motion of the ankle.

### Statistical analysis

Data was analyzed by SPSS software version 21.0. Categorical data were given as numbers and percentages, and continuous variables were presented as mean ± standard deviation or median (interquartile range-IQR), depending on the distribution of the data. The distribution of data was determined by the Kolmogorov-Smirnov test. The categorical data were compared with Chi-Square or Fisher’s Exact test. The continuous variables of the groups were compared with independent-Samples T test based on parametric and nonparametric distribution. The correlation analysis between BMI and AOFAS values was performed by Spearman’s correlation test. The continuous variables measured at different times (before and after operation) in the same patients were compared by the Wilcoxon test. A p-value less than 0.05 was considered statistically significant.

MRI imaging was performed using a 3.0 Tesla Signa Pioneer system (97 channels, GE, USA) with a 17-channel in vivo coil.

## Results

The study included 70 patients: 37 females (52.9%) and 33 males (47.1%). Median age of the patient population was noted to be 36.5 years (interquartile range: 26–49.25), while the duration of follow-up was noted to be 39 months (interquartile range: 26–54.25). The left ankle was operated on in 28 patients (40%), and the right ankle in 42 patients (60%).

The distribution of the lesions, based on the Hepple MRI classification, was described as follows: Of the cohort that was evaluated, 17 patients (24.3%) were classified as Grade 3, 49 patients (70%) were classified as Grade 4, and 4 patients (5.7%) were classified as Grade 5. Thirty-eight patients of traumatic etiology were assigned to Group 1, and 32 patients of non-traumatic etiology were assigned to Group 2. BMI analysis showed that the BMI of Group 2 was statistically significantly higher than the BMI of Group 1 (*p* = 0.0035) (Table 1). As a whole, 66 patients (94.3%) in the two groups regained the preoperative lifestyle and occupational occupation after surgery. No statistically significant difference was found between Group 1 and Group 2 in regard to return to previous levels of activity (*p* = 0.392) (Table 1).

**Table 1:** Comparative analysis of characteristics of the patients in group 1 and 2

		Group 1 (*n*=38)	Group 2 (*n*=32)	*p*
Age (year), median (IQR)		35 (25-40.5)	45.5 (31.25-51.75)	0.063
Gender, n (%)	Female	17 (44.7%)	20 (62.5%)	0.138
	Male	21 (55.3%)	12 (37.5%)	
Side, n (%)	Right	26 (68.4%)	16 (50%)	0.171
	Left	12 (31.6%)	16 (50%)	
Return to previous life, n (%)	Yes	35 (92.1%)	31 (96.9%)	0.392
	No	3 (7.9%)	1 (3.1%)	
Duration of follow-up (month), median (IQR)		43 (26-74)	36 (24.5-46.5)	0.088
Preop AOFAS score, median (IQR)		52 (49-59.5)	55 (52-61.75)	0.181
Postop AOFAS score, median (IQR)		94.5 (90-97.75)	94 (88-96.75)	0.481
Preop VAS, median (IQR)		6 (5-7)	6 (5-7)	0.658
Postop VAS, median (IQR)		0.5 (0-1)	0 (0-1)	0.406
BMI, mean±SD		25.17±2.31	26.75±3.52	0,035

*BMI* Body Mass Index, *AOFAS* American Orthopaedic Foot and Ankle Society, *VAS* Visual analogue scale, *IQR* Interquartile Range, *SD* Standart Deviation

**Fig. 3:**
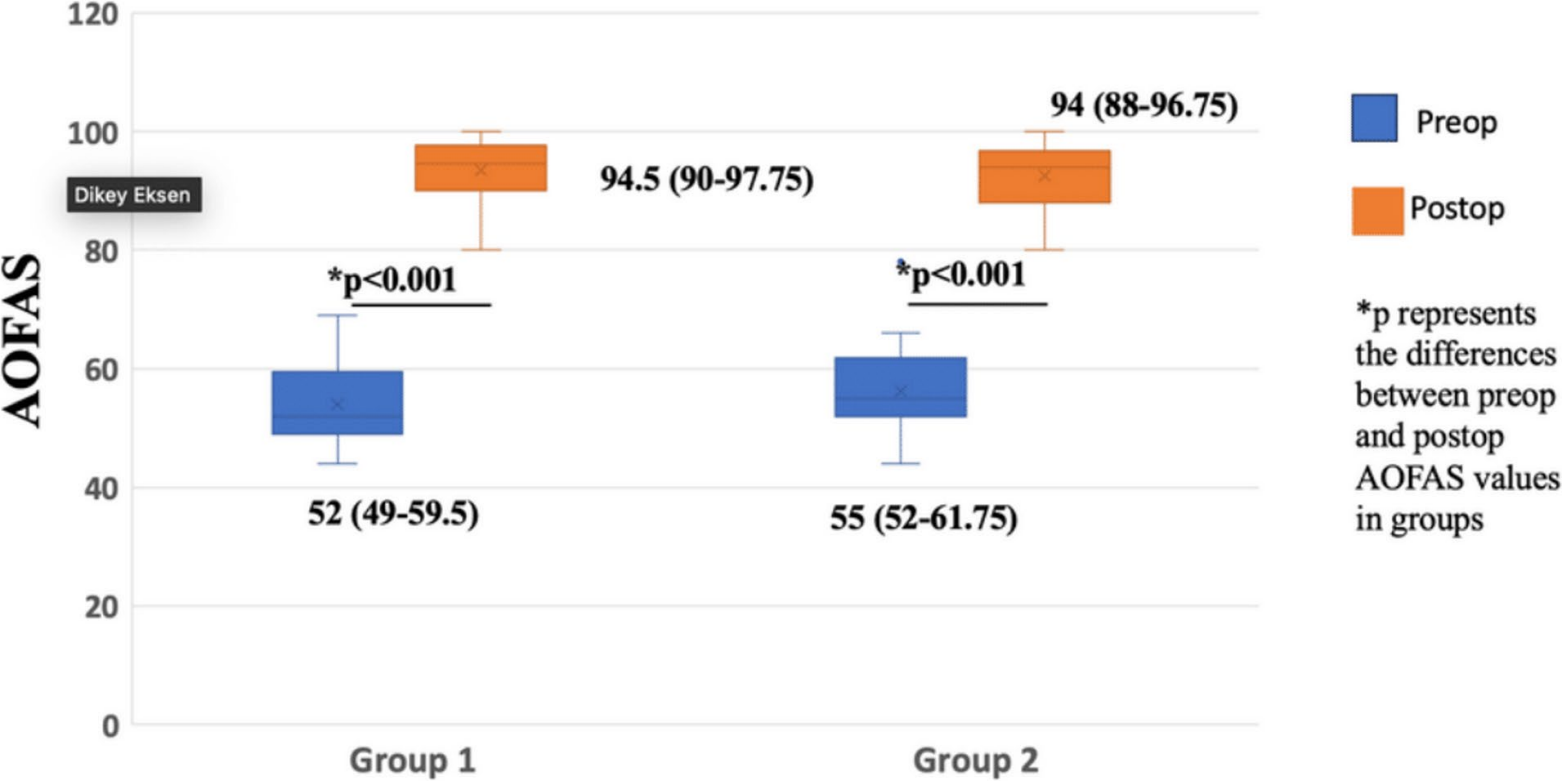
A comparison of the median (IQR) AOFAS (American Orthopaedic Foot and Ankle Society) scores before and after surgery, within and between groups. The yellow box represents Group 1 and the blue box represents Group 2. The p-values illustrate the differences seen in the median (IQR) AOFAS scores before surgery and at the end of the follow-up

Postoperative AOFAS scores showed a significant improvement in the two cohorts when compared to their preoperative scores (*p* < 0.001). A comparison of differences between Group 1 and Group 2, however, showed no significant difference in the preoperative and postoperative scores of the two groups (*p* > 0.05) (Fig. 3).

**Fig. 4:**
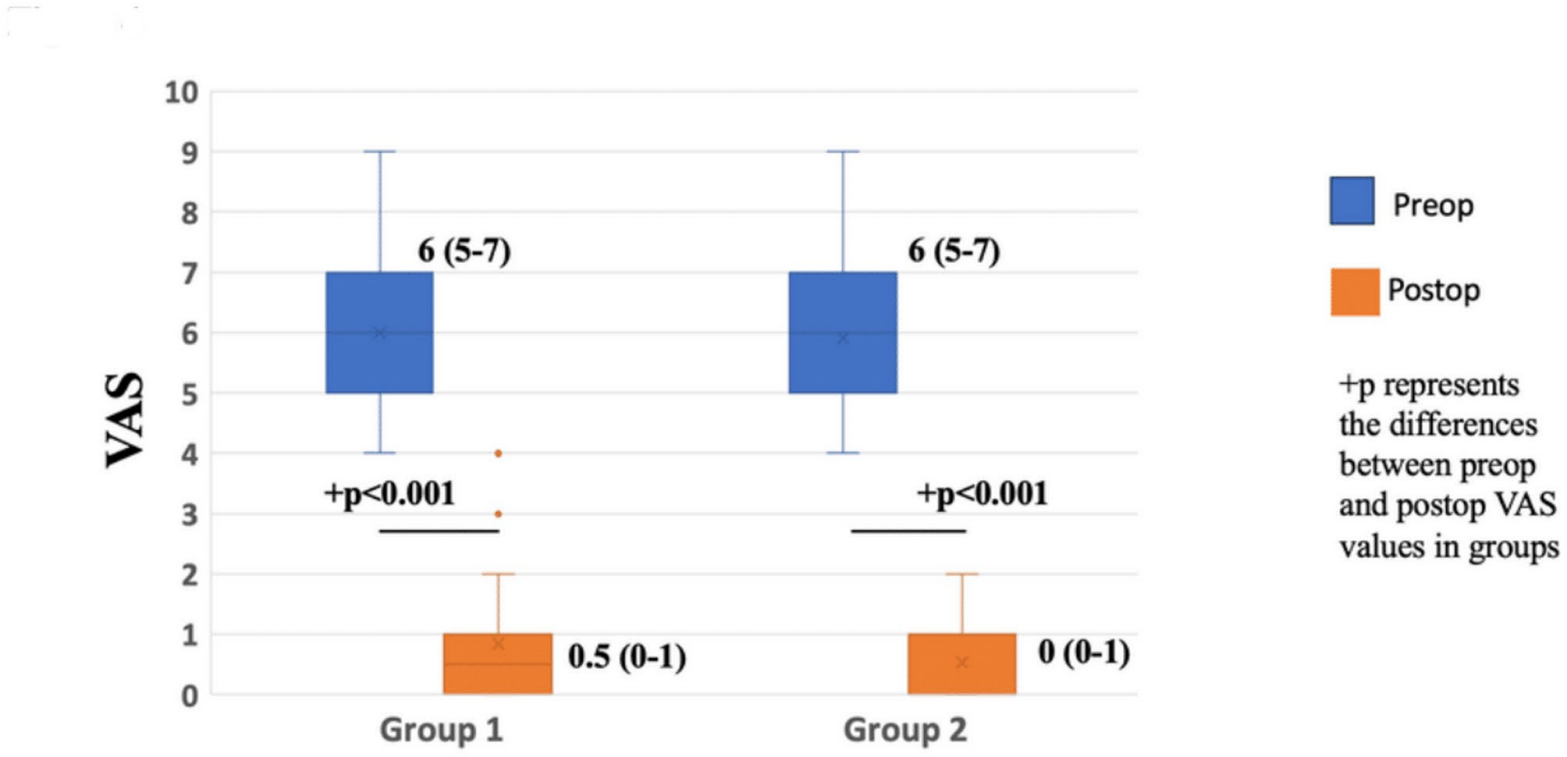
Pre- and post-operative medians (IQR) VAS (Visual analogue score) scores across the various groups. The blue represents Group 2, and the yellow represents Group 1. The p-values indicate the significance of the differences between the preoperative and postoperative medians of the VAS scores

In both groups, a p-value of < 0.001 indicates a significant improvement in the visual analog scale (VAS) scores after surgery compared to preoperative scores. No statistically significant difference was found between Group 1 and Group 2 for the preoperative median (interquartile range) VAS scores (*p* > 0.05). In addition, the postoperative median (interquartile range) VAS scores showed no differences between the groups (*p* > 0.05) (Fig. 4).

## Discussion

This study evaluated the surgical results for patients with both traumatic and non-traumatic OLTs with a median follow-up period of 39 months (interquartile range: 26–54.25 months). It concluded that clinical results after microfracture treatment were similar. The findings of this study indicate that the etiology has no significant influence on the effectiveness of the operation, suggesting that microfracture can be safely applied regardless of a patient’s history of trauma. Previous studies have found no significant influence of history of trauma on clinical results; however, these studies did not employ comparative statistics in relation to history of trauma and were limited to univariate analyses [3]. In contrast, our study directly compared traumatic and non-traumatic cases. Traumatic lesions are usually treated earlier because they present with acute symptoms such as sudden pain, swelling, loss of range of motion, and inability to bear weight on the extremity. Non-traumatic lesions, conversely, often experience delayed diagnoses due to the insidious onset of their clinical features, which may result in variable responses to treatments. Thus, etiological differences need to be taken into consideration when identifying suitable patients and developing therapeutic strategies. This study fills an essential gap in the literature by being the first to compare comprehensively the results of microfracture treatment between traumatic and non-traumatic OLTs and therefore making an essential contribution to this field.

Aside from trauma, several other predisposing factors influence the success of the microfracture technique used in the treatment of OLTs. Among them, such factors as lesion size [4, 29], depth of the lesion [30, 31], age [1, 32], BMI, history of trauma, and symptom duration have been found to have the strongest relationship with the patient’s symptom outcomes [24, 25, 29, 33]. However, some studies have opined that there is no significant difference in outcomes despite worsening of lesion size [3, 33, 34]. Additionally, lesion depth has been identified as a negative prognostic factor [30, 31]. Another study also concluded that the prognosis of OLTs is not correlated with the volume or depth of the lesion [33]. According to some studies, age may not be a significant factor in determining a poor prognosis [10]. Also, the existence of cystic formations in the vicinity of the lesion has been found to have no effect on the prognosis [35]. These findings indicate that the success of microfracture treatment depends on the morphological features of the lesion and patient-related factors, and these factors should be taken into account in treatment planning.

The bone marrow stimulation process via microfracture enables the formation of fibrocartilaginous tissue at the injury site. However, this newly developed tissue has inferior biomechanical properties compared to native hyaline cartilage [20]. With increasing dimensions of the lesion, the load-bearing capacity of the fibrocartilage decreases, thereby increasing the risk of mechanical failure [20, 24, 36, 37]. Therefore, our study was limited to patients with lesions smaller than 1.5 cm² in size. Patients with bilateral lesions were also excluded to avoid possible biases in scoring due to changed loading and healing patterns on the contralateral side.

In the current study, the follow-up period in the cohort of 70 patients was noted to be 39 months (IQR: 26–54.25), with the final AOFAS score valued at 94.5% in Group 1 and 94% in Group 2 (IQR). A previously published article demonstrated no worsening of outcomes over a 3-year period in a similar cohort of 70 patients who received microfracture [26]. Another study of 165 patients showed significant improvements over a follow-up period of 6.7 years, thus supporting microfracture as a first-line surgical procedure for OLTs in the mid-term [38]. A 10-year follow-up study reported a survival rate of 93.3% [39]. The above results demonstrate the success of microfracture, regardless of the aetiology, and suggest that most patients can return to their previous lifestyle after the procedure.

In the current study, only OLTs located within the centromedial zone (zone 4) were included. This was done to reduce any potential variations that may arise as a result of anatomical differences between different cohorts of patients and to enhance the homogeneity of our analysis with respect to surgical outcomes. OLTs are classified using a grid consisting of nine zones superimposed over the talar dome [40]. Previous literature has provided conflicting opinions regarding the prognostic significance of lesion location. Some studies have suggested that medially located lesions exhibit superior outcomes [25], while other studies have indicated that the location of the lesion has minimal or no prognostic significance [33]. Lesions located in regions corresponding to the talus shoulder have been noted to have poorer prognoses [25, 29]. By limiting the study to the centromedial zone, this study effectively eliminated anatomical variation as a confounding factor, thereby allowing for a more accurate assessment of treatment outcomes.

The mean BMI in Group 2 was 26.75 ± 3.52, a statistically significant increase compared to Group 1 (25.17 ± 2.31) (*p* = 0.0035). Some factors responsible for the development of non-traumatic OLTs include avascular necrosis, genetic predisposition, endocrine and metabolic abnormalities, repetitive microtrauma, ischemic events, and damage to vascular and synovial tissues, as well as chronic ankle instability [11, 41]. While modern literature has not been able to directly link BMI to the incidence of OLTs, it has been theorized that a high BMI could predispose to lesion formation by increasing over time the stress placed on the talus and interacting with all the contributing factors.

All patients were advised against weight-bearing after the microfracture procedure. This advice is based on the assumption that subchondral debridement and microfracture carried out in the medial surgical area compromise the structural integrity of the bone, thus increasing the chances of subsequent fractures. Some studies support this assumption, showing that avoidance of weight-bearing activities improves the quality of the newly formed tissue [42]. However, opposing studies argue that early weight-bearing on the affected limb does not negatively impact clinical results [43].

It was possible in our cohort after a period of 6 weeks without weight-bearing to achieve zero complications in the form of joint contracture, restriction of range of motion, or Sudeck’s atrophy. Based on the high degree of success and patient satisfaction, we opine that a non-weight-bearing postoperative regimen is a safe and useful regimen.

### Limitations

This research has several limitations. The retrospective nature of the study hinders the possibility of inferring causality. Prospective randomized controlled trials, coupled with multicenter and larger sample sizes, should be included in future investigations to confirm these data. Additionally, there was a lack of documentation of the physiotherapeutic treatments received by patients, which hindered the assessment of functional rehabilitation. Radiographic or clinical assessments of the evolution of osteoarthritis were not performed. Furthermore, in the non-traumatic cohort, factors other than BMI might also need to be taken into consideration.

## Conclusion

Arthroscopic microfracture treatment provides similar clinical outcomes in the case of non-traumatic and traumatic OLTs. BMI, however, has been recognized as a significant risk factor for the development of non-traumatic OLTs.

## Data Availability

The data sets generated and/or analyzed during the current study can be available from the corresponding author on a reasonable request.

## Abbreviations

OLTs: Osteocondral Lesion of Talus
MRI: Magnetic Resonance Imaging
CT: Computed Tomography
VAS: Visual Analog Scale IQR: Interquartile Range
BMI: Body Mass Index
AOFAS: American Orthopaedic Foot and Ankle Society
